# Synthetic fluorescent proteins: Bridging advanced imaging technologies and clinical practice

**DOI:** 10.1002/ctm2.70828

**Published:** 2026-09-11

**Authors:** Zhengda Chen, Xianjun Chen, Yi Yang

**Affiliations:** ^1^ Optogenetics & Synthetic Biology Interdisciplinary Research Center Shanghai Frontiers Science Center of Optogenetic Techniques for Cell Metabolism School of Pharmacy East China University of Science and Technology Shanghai China; ^2^ School of Biomedical Engineering Shanghai Jiao Tong University Shanghai China; ^3^ Shanghai Key Laboratory of New Drug Design East China University of Science and Technology Shanghai China; ^4^ Frontiers Medical Center Tianfu Jincheng Laboratory Chengdu China

1

For clinicians and clinical researchers, the ability to visualise biological processes in real‐time within living systems offers transformative potential for diagnosis, treatment monitoring and understanding disease mechanisms.^1^, ^2^ The near‐infrared (NIR) imaging window (650–900 nm) is uniquely suited for clinical imaging because it minimises light scattering, autofluorescence and haemoglobin absorption, thereby allowing deeper tissue penetration.^3^ However, due to the limited structural diversity of natural conjugated amino acids and endogenous chromophores, traditional genetically encodable NIR fluorescent proteins (FPs) are inherently constrained by trade‐offs between brightness and spectral redshift that cannot be resolved through genetic mutagenesis alone.^4^ To overcome these limitations of conventional NIR FPs, synthetic fluorescent proteins (SFPs), which combine the genetic encodability of protein tags with the superior brightness and photostability of fluorogenic dyes, have emerged as powerful tools for visualising proteins and detecting biomarkers both in vitro and in vivo.^5^ In particular, the NIR fluorogenic dyes can be coupled specifically to certain protein tags (for example, HaloTag, SNAP‐tag, FAST and DiB) to emit NIR fluorescence, and these labelling systems have been used for in vivo imaging and in vitro diagnostic assays. However, the large size of the protein tags (for example, 36 kDa for HaloTag) may be disadvantageous for tagging proteins that are spatially demanding.^6^

NirFAP680,^7^ a new NIR SFP recently developed in our laboratory, represents a significant departure from these limitations. The core achievement lies in the co‐evolution of a novel fluorogenic NIR dye, 5‐((6‐((2‐**H** ydroxyethyl)(methyl)amino) **B** enzo[b]thiophen‐2‐yl)methylene)‐3‐**M** ethyl‐2‐**T** hioxothiazolidin‐4‐one (HBMT), and a small protein tag, NirFAP. The rationally designed HBMT features good membrane permeability, low cytotoxicity, negligible background fluorescence, NIR emission (680 nm) and a remarkable Stokes shift over 100 nm. Subsequent directed evolution of mFAP2^8^ yielded NirFAP (12.6 kDa), which not only binds HBMT with high affinity (*K*_D_ = 20 nM) but also activates its fluorescence with a high quantum yield of .56. This synergistic design effectively overcomes both the brightness (<.2 quantum yield) of genetically encodable NIR FPs and the large tag size of alternative protein tags. NirFAP680 demonstrates one to two orders of magnitude greater cellular brightness than existing NIR fluorescent labels under both single‐ and two‐photon excitation, and exhibits exceptional photostability that enables long‐term super‐resolution imaging. This exceptional performance translates seamlessly to in vivo applications, as demonstrated by robust neuronal labelling in live zebrafish larvae and mouse brains via two‐photon microscopy. Beyond passive labelling, the large Stokes shift and small size make NirFAP680 an outstanding energy acceptor for fluorescence resonance energy transfer (FRET)‐ and bioluminescence resonance energy transfer (BRET)‐based biosensing. The development of a large dynamic range FRET and BRET pair makes it as an ideal candidate for live‐cell multiplexed imaging and in vitro diagnostic assays. Meanwhile, the development of an ultra‐bright NIR bioluminescent protein (NirNL) further underscores the potential of this technology for biomedical applications. Furthermore, the utility of mFAP in bimolecular fluorescence complementation systems and chemogenetic indicators, as demonstrated by Klima et al.,^9^ is also transferable to NirFAP680 by virtue of the same mechanism. (Figure 1)

**FIGURE 1:**
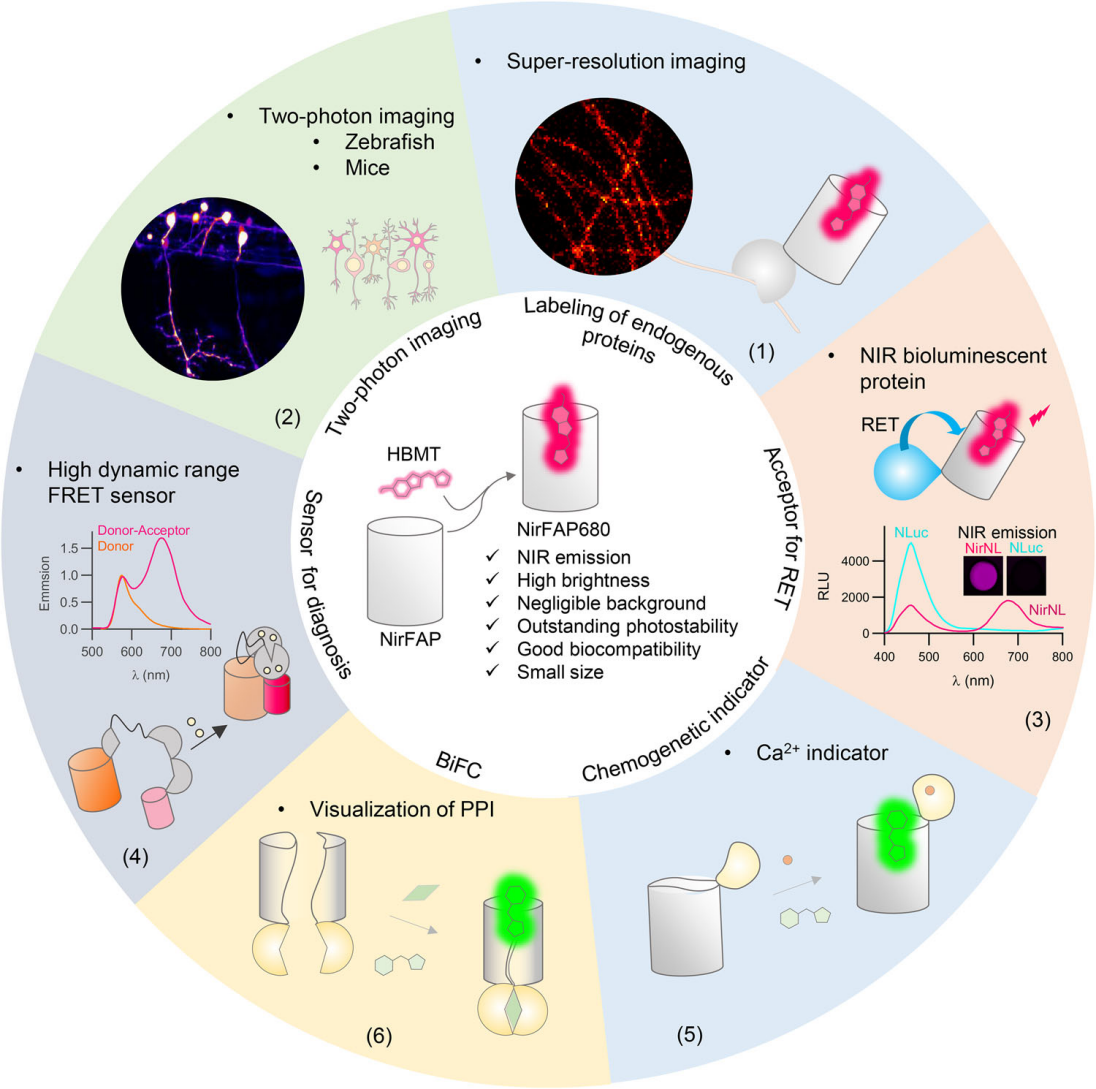
Overview of the NirFAP680 platform and its derivative imaging tools for advanced imaging. The central schematic illustrates the design and superior properties of the NirFAP680, including near‐infrared (NIR) emission, high signal background ratio and excellent photostability. The outer sectors highlight its broad utility as a versatile imaging tool: (1) a fluorescent label for super‐resolution imaging of endogenous proteins; (2) two‐photon imaging of neurons in zebrafish and mice; (3) bioluminescence resonance energy transfer (BRET)‐based high brightness NIR bioluminescent protein; (4) a high dynamic range fluorescence resonance energy transfer (FRET) sensor for diagnosis; (5) a chemogenetic indicator; and (6) a bimolecular fluorescence complementation (BiFC) system for visualising protein‒protein interactions.

Given its role as an indispensable tool for advanced imaging, NirFAP680 holds considerable translational promise and is poised to address multifaceted unmet clinical needs across several critical frontiers. First, the superior brightness and photostability of NirFAP680 make it an ideal candidate for fluorescence‐guided surgery. When combined with tumour‐associated biomarkers, this platform could enable surgeons to use NIR imaging systems to visualise and resect malignant tissues with unprecedented precision and confidence. Second, the exceptionally compact size of NirFAP680 substantially reduces the risk of altering the native function of target proteins. This feature is particularly critical for the design of clinically viable fusion constructs and biosensors, as it preserves the physiological activity of endogenous partners while enabling reliable in vivo tracking. Third, NirFAP680 and NirNL enable real‐time non‐invasive imaging of disease models at depths previously unattainable with conventional NIR FPs or luciferase. This capability allows clinicians and clinical researchers to study disease progression within physiologically relevant microenvironments, as exemplified by two‐photon imaging of neurons in live mouse brains, which offers new insights into neurological diseases. Fourth, the unique 106 nm Stokes shift of NirFAP680 enables the construction of high‐sensitivity FRET‐ or BRET‐based systems, which are particularly valuable for real‐time detection of protein‒protein interactions and signalling pathway activity. This sensitivity is crucial for developing ultra‐sensitive diagnostic assays for a wide range of diseases, where early and reliable detection of biomarkers is paramount. Collectively, these advantages establish NirFAP680 as a transformative asset for translational medicine, with the potential to influence how clinicians visualise, diagnose and treat diseases—ultimately bringing the promise of NIR imaging closer to the bedside.

Despite these exciting prospects, the path to clinical translation of any new imaging agent requires careful consideration. First, the fluorogen HBMT must undergo rigorous toxicological evaluation and regulatory approval for human use, including assessments of its pharmacokinetics, tissue clearance and potential immunogenicity. Second, delivery methods, whether via viral vectors, mRNA, or protein transduction, require optimisation for safety and efficiency in patients, with particular attention to targeting specificity, expression durability and scalability for clinical manufacturing. Third, imaging hardware compatible with NirFAP680's excitation and emission spectra must be widely available in clinical settings, which will necessitate both hardware upgrades in existing surgical and endoscopic systems and the training of medical personnel to interpret NIR fluorescence signals accurately. Nevertheless, the rapid pace of innovation in NIR imaging hardware and the growing acceptance of fluorescence‐guided interventions in surgery and endoscopy make the clinical adoption of NirFAP680 a realistic near‐term goal. Realising this goal will depend not only on technological readiness but also on effective interdisciplinary collaboration; close partnerships between basic scientists, bioengineers and clinicians will be essential to translate this technology from bench to bedside.

In summary, the development of NirFAP680 represents a significant leap forward in advanced imaging, effectively bridging the gap between fundamental discovery and potential clinical application. By providing a bright, stable and versatile NIR imaging tool, this technology not only overcomes the inherent limitations of previous NIR FPs but also opens up exciting new possibilities for visualising and understanding disease processes in their native, complex environments. We anticipate that NirFAP680 will catalyse a new wave of translational research, moving us closer to the realisation of truly personalised and image‐guided medicine.

## AUTHOR CONTRIBUTIONS

Z.C., X.C. and Y.Y. wrote the manuscript. All authors reviewed and approved the final version of the manuscript.

## CONFLICT OF INTEREST STATEMENT

All the authors declare they have no conflicts of interest.

## ETHICS STATEMENT

All animal experiments must conform to relevant national and institutional regulations. The animal study protocols were approved by the Institutional Animal Care and Use Committee of East China University of Science and Technology.

## References

[1] Wang K , Du Y , Zhang Z , et al. Fluorescence image‐guided tumour surgery. Nat Rev Bioeng. 2023;1:161‐179. doi:10.1038/s44222‐022‐00017‐1

[2] Li YC , Chen QH , Pan XY , Lu W , Zhang J . New insight into the application of fluorescence platforms in tumor diagnosis: from chemical basis to clinical application. Med Res Rev. 2023;43:570‐613. doi:10.1002/med.21932 36420715 10.1002/med.21932

[3] Shcherbakova DM , Stepanenko OV , Turoverov KK , Verkhusha VV . Near‐infrared fluorescent proteins: multiplexing and optogenetics across scales. Trends Biotechnol. 2018;36:1230‐1243. doi:10.1016/j.tibtech.2018.06.011 30041828 10.1016/j.tibtech.2018.06.011 PMC6240479

[4] Zhang H , Papadaki S , Sun X , et al. Quantitative assessment of near‐infrared fluorescent proteins. Nat Methods. 2023;20:1605‐1616. doi:10.1038/s41592‐023‐01975‐z 37666982 10.1038/s41592-023-01975-z PMC11753454

[5] Porzberg N , Gries K , Johnsson K . Exploiting covalent chemical labeling with self‐labeling proteins. Ann Rev Biochem. 2025;94:29‐58. doi:10.1146/annurev‐biochem‐030222‐121016 40106680 10.1146/annurev-biochem-030222-121016

[6] Frei MS , Tarnawski M , Roberti MJ , Koch B , Hiblot J , Johnsson K . Engineered HaloTag variants for fluorescence lifetime multiplexing. Nat Methods. 2022;19:65‐70. doi:10.1038/s41592‐021‐01341‐x 34916672 10.1038/s41592-021-01341-x PMC8748199

[7] Chen Z , Jiang Li , Liu X , et al. NirFAP680: a highly bright and stable large Stokes shift near‐infrared fluorogen‐activating protein. Nat Methods. 2026;23:960‐971. doi:10.1038/s41592‐026‐03061‐6 42014878 10.1038/s41592-026-03061-6

[8] Dou J , Vorobieva AA , Sheffler W , et al. De novo design of a fluorescence‐activating β‐barrel. Nature. 2018;561:485‐491. doi:10.1038/s41586‐018‐0509‐0 30209393 10.1038/s41586-018-0509-0 PMC6275156

[9] Klima JC , Doyle LA , Lee JD , et al. Incorporation of sensing modalities into de novo designed fluorescence‐activating proteins. Nat Commun. 2021;12:856. doi:10.1038/s41467‐020‐18911‐w 33558528 10.1038/s41467-020-18911-w PMC7870846

